# Impact of alcohol use disorder on inpatient hospitalizations: A comparison of outcomes between urban and rural Veterans Affairs hospitals

**DOI:** 10.1002/jhm.13544

**Published:** 2024-12-02

**Authors:** James Willey, Peter Kaboli, Andrea Holcombe, Amy M. J. O'Shea, Tong Yu, Kelby Rewerts, George Bailey, Anindita Bandyopadhyay, Thad Abrams, Jeydith T. Gutierrez

**Affiliations:** ^1^ Comprehensive Access and Delivery Research and Evaluation (CADRE) Center Iowa City VA Healthcare System Iowa City Iowa USA; ^2^ VA Office of Rural Health, Veterans Rural Health Resource Center ‐ Iowa City (VRHRC‐IC) Iowa City Veterans Affairs Health Care System Iowa City Iowa USA; ^3^ Department of Internal Medicine University of Iowa Iowa City Iowa USA; ^4^ Department of Internal Medicine University of Utah Salt Lake City Utah USA; ^5^ Department of Psychiatry University of Iowa Iowa City Iowa USA

## Abstract

**Background:**

Alcohol use disorder (AUD) is a leading cause of morbidity and mortality that disproportionately affects rural residents and Veterans.

**Objective:**

To evaluate the burden of AUD in admissions at rural and urban hospitals within the Veterans Health Administration (VHA) comparing patient characteristics, clinical outcomes, and 1‐, 3‐, and 5‐year mortality rates.

**Methods:**

Retrospective cross‐sectional study of patients admitted to VHA hospitals from 2016 to 2020, with a primary or secondary diagnosis related to AUD. Follow‐up mortality data was collected through September 30, 2023.

**Results:**

From 2.9 million qualifying admissions, AUD‐related diagnoses were present in 14.3% of admitted patients (427,375 admissions among 190,152 unique patients in 129 facilities). Rural hospitals (*n* = 22) had a significantly higher overall admission rate for AUD‐related diagnoses (21.6% vs. 14.8%, *p* = .011), higher 30‐day readmission rates (17.8% vs. 15.3%, *p* < .001), but lower hospital‐level average length of stay (median = 4.3 vs. 5.6, *p* < .001). Mortality in rural hospitals was lower than urban at 1 year (9.6% vs. 11.4%, *p* < .001), 3 years (20.7% vs. 23.1%, *p* < .001), and 5 years (30.4% vs. 32.9%, *p* < .001).

**Conclusions:**

Rural VHA hospitals have a higher proportion of patients admitted with AUD‐related diagnoses and higher readmission rates, but lower mortality rates. Approximately, one in three patients admitted with an AUD‐related diagnosis died within the 5‐year follow‐up period. The mortality rates observed are extraordinary and deserve urgent attention. A comprehensive plan to address AUD in the Veteran population, including how we approach and engage patients in treatment during hospitalizations with any primary or secondary AUD diagnoses, is needed.

## Visual Abstract

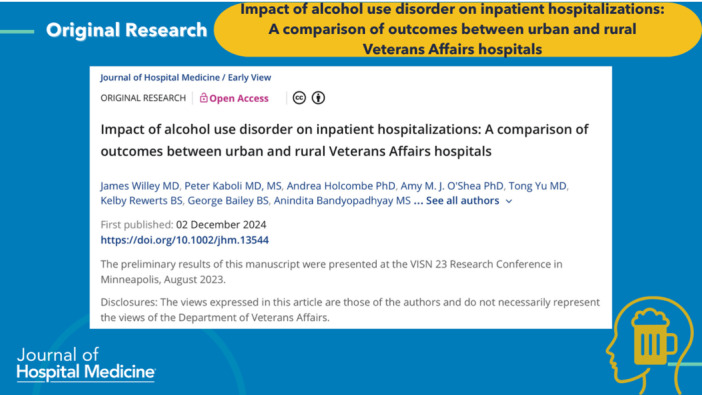

## INTRODUCTION

Alcohol use disorder (AUD) is a medical condition characterized by an inability to stop or control alcohol use despite adverse social, occupational, or health consequences.^1^ AUD is a leading cause of morbidity and mortality in the United States, resulting in ~140,000 preventable deaths annually.^2^, ^3^ Prevalence of AUD in the United States is estimated to be about 5.3% for people 12 years or older, but disproportionately affects Veterans and rural communities.^4^ Rural Americans have a higher alcohol‐induced death rate among adults aged 25 and older.^5^ Veterans are more likely to report alcohol use (56.6% vs. 50.8%) and binge drinking (7.5% vs. 6.5%) in the previous month compared with non‐Veteran counterparts.^6^

Data show that both AUD incidence and AUD‐related hospitalizations are increasing, with the United States recording over 2.6 million admissions with any alcohol‐related diagnosis in 2015.^7^, ^8^ An AUD diagnosis increases acute care service utilization (e.g., emergency department‐visits, 30‐day readmissions) and is associated with higher in‐hospital mortality, sepsis, hospital acquired infections, and length of stay (LOS).^9^, ^10^, ^11^ In an european study a diagnosis of AUD was associated with a 24–28 years shorter lifespan.^12^ Despite the high cost, morbidity and mortality associated with AUD, there is a paucity of studies assessing outcomes in hospitalized patients, and no studies on long‐term outcomes on hospitalized Veterans.

Our study objective was to assess hospital burden and clinical outcomes of patients admitted with any primary or secondary AUD‐related diagnosis within the Veterans Health Administration (VHA) stratified by hospital rurality. We described patient and facility characteristics and clinical outcomes (LOS, 30‐day, and 1‐, 3‐, and 5‐year mortality, and 30‐day readmission rate) among both groups.

## METHODS

### Study design

This was a retrospective cross‐sectional study of a national VHA data set approved by the University of Iowa Institutional Review Board and the Iowa City VA Healthcare System Research and Development Committee. STROBE reporting guidelines for epidemiologic observational studies were followed.^13^

### Setting

For this study, we considered a cohort of patients treated at 172 Veterans Affairs (VA) hospitals between fiscal year (FY) 2016 and FY 2020 (i.e., October 1, 2015, through September 30, 2020). We excluded 43 hospitals without any inpatient beds, resulting in 129 hospitals (107 urban and 22 rural) in the final study cohort. To account for inpatient service variations over time, the number of medical and surgical beds per FY for each facility was assessed. Facilities without inpatient medical or surgical beds during any given study FY had that year's data excluded.

### Cohort, data variables, and outcome definitions

Eligible admissions included those where the patient was admitted to inpatient or observation status in psychiatry, medicine, noncount (undefined service), or surgery services. For FY 2016–2020, all VHA inpatient and observation status admissions were reviewed for any AUD‐related diagnosis. AUD‐related diagnosis were defined as the presence of any primary or secondary diagnosis with alcohol‐related International Classification of Diseases, 10th revision (ICD‐10) codes during the hospitalization (Supporting Information S1: Table 1).^14^, ^15^ Admissions without an AUD‐related diagnosis were excluded. For each patient, we identified the first admission with any AUD‐related diagnosis during the study period.

Patient characteristics are described at the time of the first AUD‐related admission. Patient‐level variables included age, Elixhauser Comorbidity Index score, rurality, sex, race, and ethnicity. ICD‐10 codes for conditions included in the Charlson and Elixhauser Comorbidity Indexes were collected from patients' problem lists (Supporting Information S1: Table 2). Mortality data spanned October 1, 2015, through September 30, 2023. Mortality rates were calculated from the time of first admission with AUD‐related diagnosis to 1‐, ‐3‐, and 5‐years after first admission. The denominator included all patients with 1‐, 3‐, and 5‐years between the first admission and our cutoff of September 30, 2023

Clinical profiles were created using the VHA Corporate Data Warehouse (CDW), including admission dates, ICD‐10 codes for primary and secondary diagnoses, admitting service, and mortality data. This information was used to calculate the 30‐day mortality rate, hospital LOS, and 30‐day readmission rates. A readmission was characterized as an admission to the discharging hospital or to any VHA hospital occurring at least 12 h and up to 30‐days after the first admission's discharge. Re‐admissions within 12 h of the first discharge were removed as duplicates, often caused by changes in level of care (e.g., conversion from observation to inpatient). We obtained data on overall yearly admissions per hospital and calculated the hospital‐level proportion of admissions with concurrent AUD‐related diagnosis. AUD admission rates were calculated as the ratio of the total number of AUD admissions over total admissions during the study period for each hospital. Similarly, 30‐day mortality and 30‐day readmission rates were calculated as the ratio of events over total AUD admissions by hospital during the study period. The LOS for each AUD admission during the study period was calculated and averaged within hospital.

Hospital‐level characteristics included the facility complexity level, treating speciality, and highest level of care during the admission. Complexity level ranks VHA facilities on a scale from 1a to 3, with 1a representing the highest level of complexity and 3 signifying the lowest. Low‐complexity hospitals are those with the lowest volume and patient complexity levels and minimal or no teaching programs, research, intensive care unit (ICU) beds, or subspecialists. Highest level of care for each admission was defined as the highest level of care that a patient had at any point between the admission and discharge date, with ICU being the highest and observation being the lowest.

### Analyses

We summarize patient‐, admission‐, and hospital‐level characteristics as means or proportions overall and stratified by hospital rurality. Mean admission, 30‐day mortality, and 30‐day readmission rates, as well as the median of the hospital‐averaged LOS are provided. Differences in the distribution of patient characteristics, hospital characteristics, and outcomes were evaluated using *χ* ^2^ tests, *t* tests, or Wilcoxon rank sum test as appropriate. A sensitivity analysis was performed with all observation admissions removed to allow comparison to other studies which might have excluded observation status admissions (Supporting Information S1: Table 3). A caterpillar plot of yearly AUD‐related diagnoses admission rates by hospital and categorized by hospital rurality is provided. A frailty model with hospital as the random effect evaluated the association between AUD patient survival rate and the rurality of the patient's first admitting hospital, adjusted for age, race, sex, and patient rurality.

Given that our enrollment and follow‐up period included years during the COVID‐19 pandemic, we conducted two sensitivity analyses (1) where the study enrollment and 1‐year follow‐up ended in February 2020 (before the COVID‐19 pandemic) and (2) evaluated 1‐ and 3‐year mortality among patients with first hospitalization occurring between March and September 2020.

The authors had full access to and take full responsibility for the integrity of the data. All analyses were conducted using R version 4.1.2 and SAS software version 9.4 (SAS Institute).

## RESULTS

### Patient and hospital characteristics

**Figure 1:**
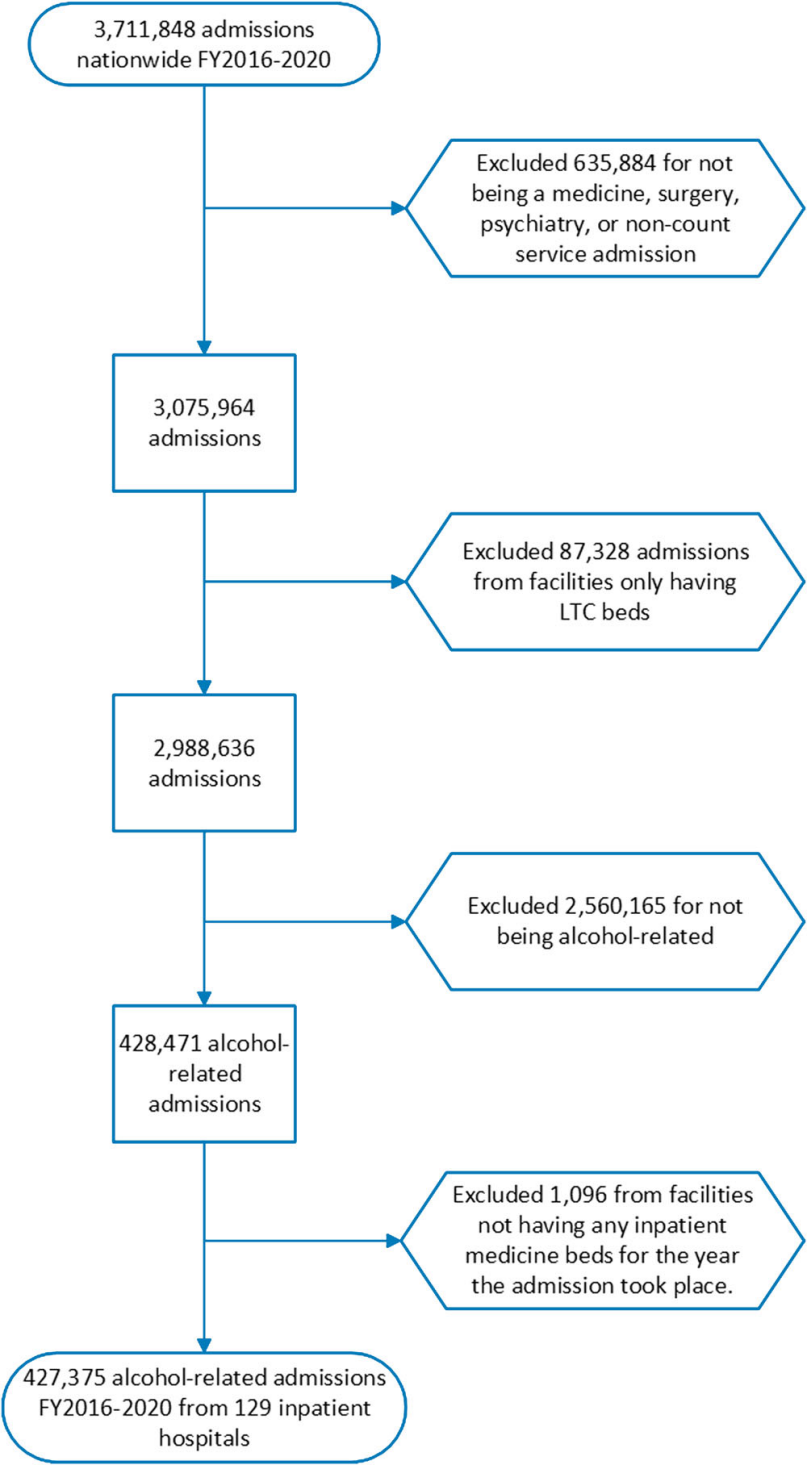
Flowchart of cohort creation for AUD‐related admissions from VHA data, FY2016–2020. [Correction added on 19 April 2025, after first online publication: The caption of figure has been revised.]

From 2.9 million admissions, AUD‐related diagnoses were identified in a total of 427,375 admissions (14.3%) among 190,152 unique patients during the study period (Figure 1). Patients admitted to rural hospitals were younger (55.4 vs. 57.5 years old, *p*<.001), more likely to identify as white (82.7% vs. 63.9%), and non‐Hispanic (94.7% vs. 89.2%, *p*<.001) than those admitted to urban hospitals (Table 1).

**Table 1:** Descriptive statistics and unadjusted comparisons of unique patients admitted with AUD‐related diagnoses in rural versus urban VA hospitals, FY2016–FY2020.

	All hospitals *N* = 129	Rural hospitals^a^ *N* = 22	Urban hospitals *N* = 102	*p* Value^b^
Number of patients	**190,152**	**12,953**	**177,199**	
Age at first admission, mean (SD)	57.3 (14.0)	55.4 (14.1)	57.5 (14.0)	<.001
Elixhauser score at first admission, mean (SD)^c^	1.3 (1.46)	1.4 (1.49)	1.3 (1.46)	<.001
Rurality (patient address)				<.001
Urban	138,937 (73.1)	5045 (39.0)	133,892 (75.6)	
Rural	49,317 (25.9)	7517 (58.00)	41,800 (23.6)	
Highly rural	1691 (0.9)	378 (2.9)	1313 (0.7)	
Missing	207 (0.1)	13 (0.1)	194 (0.1)	
Sex				.047
Male	180,479 (94.9)	12,342 (95.3)	168,137 (94.9)	
Female	9673 (5.1)	611 (4.7)	9062 (5.1)	
Race				<.001
American Indian or Alaskan Native	2333 (1.2)	378 (2.9)	1955 (1.1)	
Asian, Native Hawaiian, or Pacific Islander	2121 (1.1)	92 (0.7)	2029 (1.2)	
Black or African American	49,933 (26.3)	1150 (8.9)	48,783 (27.5)	
White	11,895 (6.3)	10,706 (82.7)	113,164 (63.9)	
Declined/unknown/missing	12,3870 (65.1)	627 (4.8)	11,268 (6.4)	
Ethnicity				<.001
Hispanic or Latino	12,470 (6.6)	214 (1.7)	12,256 (6.9)	
Not Hispanic or Latino	170,288 (89.6)	12,267 (94.7)	158,021 (89.2)	
Declined/unknown/missing	7394 (3.9)	472 (3.6)	6922 (3.9)	
Mortality rate				
1‐year, rate (%)	21,514/190,152 (11.3)	1239/12,953 (9.6)	20,275/177,199 (11.4)	<.001
3‐year, rate (%)	43,561/190,082 (22.9)	2685/12,949 (20.7)	40,876/177,133 (23.1)	<.001
5‐year, rate (%)	42,682/130,508 (32.7)	2818/9280 (30.4)	39,864/121,228 (32.9)	<.001

a Rural and urban hospital is designated by the hospital at which the patient was first admitted in study period.

^b^ Categorical variables were assessed via *χ* ^2^ Test, while numeric variables were assessed using the two‐sample *t* test. All hypothesis tests were two‐sided.

^c^ Patients without an identified Elixhauser score condition were assumed to not have these conditions, see Supporting Information S1: Table 2.

**Figure 2:**
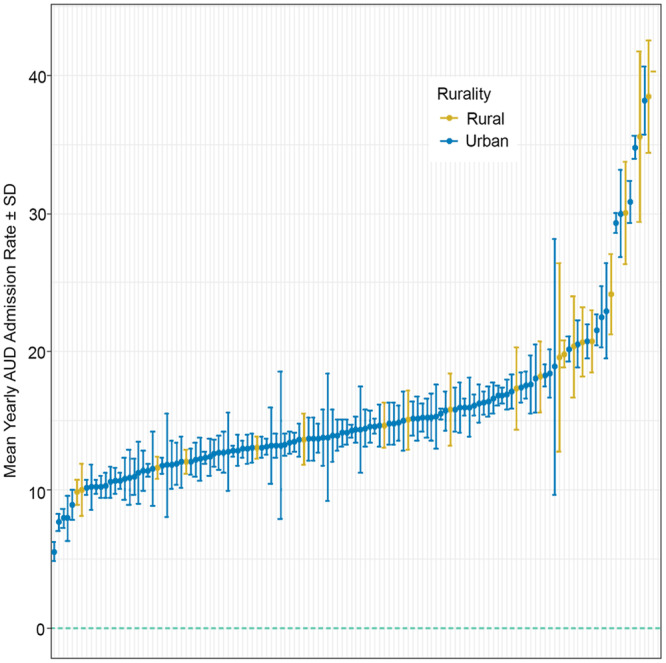
Mean yearly number of AUD‐related admissions among 129 VA hospitals (ordered by percentage of AUD admits smallest to largest).

Admissions with AUD‐related diagnosis represented 17.8% (*n* = 31,580) and 14.0% (*n* = 395,795) of total admissions at rural and urban hospitals, respectively, while average yearly admissions with AUD‐related diagnoses were 21.6% and 14.8%, respectively (Table 2). AUD‐related diagnosis admission rates varied both within and between rural and urban hospitals (Figure 2). Notably, the facilities with the highest average yearly AUD‐related diagnosis admission rates were rural (Table 2).

**Table 2:** Descriptive statistics and unadjusted comparison of hospital‐level characteristics of AUD‐related admissions in rural versus urban VA hospitals, FY2016–FY2020.

	All hospitals (*N* = 129)	Rural hospitals (*N* = 22)	Urban hospitals (*N* = 107)	*p* Value^a^
Percentage of AUD‐related admissions out of all admissions, % (*n*)	14.3 (427,375)	17.8 (31,580)	14.0 (395,795)	<.001
AUD‐related admissions yearly rate, mean % (SD)	16.0 (6.9)	21.6 (11.3)	14.8 (5.0)	.011
30‐day mortality rate, mean % (SD)	2.9 (1.3)	2.0 (0.8)	3.1 (1.3)	<.001
Average LOS, days, median (IQR)	5.4 (4.6–6.4)	4.3 (3.4–5.6)	5.6 (4.7–6.5)	<.001
30‐day readmission rate, mean % (SD)	15.8 (4.1)	17.8 (4.9)	15.3 (3.8)	<.001
30‐day readmission rate to any VHA hospital, mean % (SD)	17.2 (4.2)	19.6 (5.3)	16.7 (3.8)	.026
Number of hospitals by complexity, *n* (%)^b^				<.001
1a, high complexity	42 (32.8)	1 (4.6)	41 (39.6)	
1b, high complexity	21 (16.4)	0 (0.0)	21 (19.8)	
1c, high complexity	23 (12.5)	2 (9.1)	21 (19.8)	
2, medium complexity	22 (17.2)	9 (40.9)	13 (12.3)	
3, low complexity	20 (15.6)	10 (45.5)	10 (9.4)	
Admission level data, total admission *N*	*N* = 427,375	*N* = 31,580	*N* = 395,795	
Treating specialty, *n* (%)				<.001
Medicine	20,8877 (48.9)	19,048 (60.3)	189,829 (48.0)	
Psychiatry	127,679 (29.9)	7851 (24.9)	119,828 (30.3)	
Surgery	26,540 (6.2)	242 (0.8)	26,298 (6.6)	
Noncount	64,279 (15.0)	4439 (14.1)	59,840 (15.1)	
Highest level of care, *n* (%)				<.001
Intensive care unit	34,387 (8.1)	1999 (6.3)	32,388 (8.2)	
Inpatient	353,485 (82.7)	27,004 (85.5)	326,481 (82.5)	
Observation	39,503 (9.2)	2577 (8.2)	36,926 (9.3)	

a Categorical variables were assessed via *χ* ^2^ Test. Rates were assessed using the two‐sample *t* test, and medians were assessed via the Wilcoxon Rank Sum Test. All hypothesis tests were two‐sided.

^b^ One urban hospital has a complexity level identified as excluded due to being a joint DOD/VA hospital and is not included in these totals. [Correction added on 19 April 2025, after first online publication: The table has been revised]

Median hospital‐averaged LOS was 4.3 days in rural hospitals and 5.6 days in urban hospitals, while the average 30‐day readmission rate was 17.8% in rural hospitals and 15.3% in urban hospitals. Primary team specialty for admissions was predominantly medicine in both rural and urban hospitals (60.3% vs. 48.0%), although psychiatry (24.9% vs. 30.3%) and surgery (0.8% vs. 6.6%) had a larger proportion of patients with AUD diagnoses in urban hospitals. ICU utilization as a percentage of total admissions was lower in rural than in urban centers (6.3% and 8.2%), as was observation admissions (8.2% vs. 9.3%).

### Mortality

Average 30‐day all‐cause mortality in AUD patients admitted to rural hospitals was lower than those admitted to urban hospitals (2.0% vs. 3.1%, *p* < .001). (Table 2) This mortality difference persisted beyond 30 days with rural versus urban hospitals registering 1‐year (9.6% vs. 11.4%), 3‐year (20.7% vs. 23.1%), and 5‐year (30.4% vs. 32.9%) mortality rates, respectively (Table 1). The results of the frailty model, used to evaluate the association between AUD patient survival and facility rurality, did not indicate evidence that hospital rurality was a risk factor (Supporting Information S1: Table 4). However, Native American/Alaskan Indian (compared with White) and age at first admission were associated with an increased mortality rate. Female sex, patient rurality, Asian/Native Hawaiian/Pacific Islander race, and black race (compared with White) were associated with a lower mortality rate at 5‐years.

### COVID‐19 sensitivity analyses

Out of the 427,378 admissions included in the study, 43,516 (10.2%) occurred after the beginning of the COVID‐19 pandemic, during March–September, 2020. From October 2020 to the study end, 14,229 (7.5%) Veterans in the study had their first admissions with an AUD‐diagnosis. Analyses of this subset of patients showed that they had a 12.9% (10.7% rural, 13% urban) 1‐year and 24.3% (24.4% rural, 24.3% urban) 3‐year mortality rate (Supporting Information S1: Table 5). This was slightly higher than the overall rate of 11.3% (9.6% rural, 11.4% urban) at 1‐year and 22.9% (20.7% rural and 23.1% urban) at 3‐year for our overall cohort.

## DISCUSSION

In this study, we sought to compare outcomes of patients admitted to rural versus urban VA hospitals with the presence of any AUD‐related diagnoses. There is, to our knowledge, no comparable studies on the burden of AUD among hospitalized Veterans, or previous long‐term studies examining the impact of AUD diagnoses on hospital admissions outcomes in the United States. We found that the percentage of total admissions with AUD diagnoses in our cohort of over 2.9 million admissions was 14.3% overall. This is much higher than the previously reported prevalence of 5.8% (4.1%–7.3%) of AUD among hospital admissions from 2014 to 2018 in the US population.^16^ Our higher prevalence is partly explained by cohort identification, given that the NHAMCS survey data used a narrower set of ICD‐9 codes in this national study. Yet, they also included any mention on the chart of terms that suggested the presence of AUD *(i.e: alcoholism, excessive alcohol use, heavy drinking, etc.)*, which we did not. Prevalence of AUD in Veterans and rural residents is known to be higher than in the general population.^4^, ^5^ Further, VA hospitals' cultural practices and available resources for substance use treatment may explain the higher prevalence and/or reporting than in the general population. Lower thresholds for admission of patients with AUD might also be influenced by the VHA's focus on preventing Veteran suicide known to be associated with AUD.^17^ Our results add to the literature by describing the magnitude of the impact that AUD has on hospitalized Veterans and the differences between rural and urban VA hospitals.

With regard to the difference between urban and rural centers, admissions with AUD‐ related diagnoses out of all admissions accounted for 21.6% per year averaged in rural VA hospitals and 14.8% in urban VA hospitals, with some rural facilities having a yearly average as high as 30% of their admissions with AUD diagnoses. Patients admitted to rural hospitals were less likely to be admitted to the ICU, had lower 30‐day, and 1‐, 3‐, and 5‐year mortality, but higher readmission rates compared with those admitted to urban hospitals. Higher mortality in urban hospitals was expected, based on the higher ICU admission rates and older age. Furthermore, increased mortality at urban hospitals might be due to the concentration of higher acuity patients at larger urban centers. Thirty‐day readmission rates for patients with AUD diagnoses were 17.8% at rural and 15.3% at urban hospitals. We suspect that higher rates of admissions and readmissions in rural VA hospitals are a reflection of multiple factors, including lower thresholds for admissions and higher bed availability, limited access to substance abuse treatment resources and outpatient programs, and possibly due to higher prevalence of AUD in rural areas.

A key finding that deserves emphasis is the 5‐year mortality rate of over 30% in both rural and urban hospitals. Although no previous large US‐based studies have reported on long‐term outcomes of patients admitted with AUD diagnoses, these mortality rates were much higher than prior reports from European studies. A Danish study evaluating hospital mortality after 15 years of first‐time hospital contact with an alcohol problem, showed a 1‐year mortality rate of 3.8% for women and 5.2% for men, and a 5‐year mortality rate of 14% for women and 16% for men.^18^ There are probably many reasons for the differences seen in our 5‐year mortality rates and the ones reported in this Danish study. First, the Danish study enrolled patients at first contact ever, whereas we enrolled patients admitted at any time during our study period and therefore we have an older patient cohort. We also used a larger catchment of patients with any AUD diagnosis, and there is a known higher prevalence of AUD in Veterans. Furthermore, there are Veteran‐specific characteristics that might put Veterans at higher risk of poor outcomes (i.e., increased burden of mental health diagnoses, co‐occurring substance use, older age, and other comorbidities). Additional studies to further characterize and explain the high mortality rates in this population and long‐term studies of AUD effects in US non‐Veteran populations are needed. Similar to our conclusions, the Danish study suggested that prioritizing interventions to reduce harmful alcohol consumption among hospitalized patients with AUD diagnoses could result in substantial reduction on healthcare costs, subsequent hospitalizations, and premature mortality.

Given that our study partially took place during the COVID‐19 pandemic, we acknowledge that COVID‐19 probably played a role in the overall increased mortality, and could have affected rates of admissions and readmissions. It is known that COVID‐19 disrupted access to care in general, and although the VHA rapidly pivoted to providing care via telehealth, we hypothesized that the pandemic could have influenced patient's and provider's behaviors, impacting study results. We presume that this impact is modest, and the high mortality rates described, can not be attributed to the COVID‐19 pandemic alone. Mortality for our cohort admitted with AUD diagnoses was highest within the first and second year after the index admission and decreased in years 3–5. The majority (75.6%) of our cohort had a first admission that occurred at least 1‐year before March 2020. In addition, a subset analysis of patients whose first admission occurred between March and September of 2020, showed only a slight increase in 1‐ and 3‐year mortality rate compared with the overall cohort (12.9% vs. 11.3% and 24.3% vs. 22.9%, respectively). Although other studies have shown AUD morbidity and mortality worsened during the COVID‐19 pandemic, with an associated rise of alcohol related deaths by 7.1%, our study was not designed to assess the specific impact of COVID‐19.^19^ Future studies to better understand causes of death within this cohort might be better suited to quantify the effect of the pandemic on the high mortality rates observed in this study.

This study has a number of limitations. First, these results might not be generalizable to non‐Veteran populations, as it is possible that certain Veteran characteristics put them at higher risk of poor outcomes. Second, the use of ICD‐10 billing codes to identify the primary or secondary diagnosis of AUD could result in an inaccurate estimation of the prevalence, based on completeness of the medical record and documentation. Third, hospital admission practices based on local resources might vary—hospital admissions might be avoided based on local availability of outpatient alcohol detox programs, local inpatient rehabilitation centers, and addiction specialist providers to facilitate outpatient detox. Urban hospitals often have greater access to addiction specialists and addiction specialty clinics, which may lead to some of the discrepancies we found between rural and urban centers. Fourth, this study does not capture Veterans who receive care outside of the VHA system, and likely underestimates the true burden of AUD in both rural and urban centers. Fifth, mortality in this study is evaluated only from the initial enrollment admission and does not account for other risk factors that might have developed with readmissions or switch from hospital type (urban/rural) during the follow‐up period. Finally, reasons why patients were admitted or readmitted could be further explored in additional research to understand how other factors play a role in the elevated mortality of these patients.

## CONCLUSIONS

VHA hospitals have a high burden of patients admitted with concurrent AUD diagnosis, with rural hospitals having a disproportionate percentage compared with urban hospitals. The high burden of AUD at rural VA hospitals should be a national target for a VA intervention to mobilize addiction medicine resources, either through local program development, cross‐state addiction programs, or telemedicine initiatives. From our analysis, we highlight that AUD diagnosis in hospital admissions confers a mortality risk on par with mortality rates for localized lung cancer diagnosis.^20^ The mortality rates observed are extraordinary and deserve urgent attention. A comprehensive plan to address AUD in the Veteran population, including how we approach and engage patients in treatment during hospitalizations with an identified AUD diagnosis, and how to establish appropriate referrals and follow‐ups, is needed.

## CONFLICT OF INTEREST STATEMENT

The authors declare no conflict of interest.

## Supporting information

Supporting information.

## Data Availability

Data supporting the findings of this study are available on request from the corresponding author. The data are not publicly available due to privacy or ethical restrictions.
