# Supplementary material for: Impact of alcohol use disorder on inpatient hospitalizations: A comparison of outcomes between urban and rural Veterans Affairs hospitals
Source: J Hosp Med. 2024 Dec 2;20(5):463–70. doi: 10.1002/jhm.13544 (PMC12047202; doi:10.1002/jhm.13544)
Supplement: Supplementary file 1 — Supporting information. [file JHM-20-463-s001.docx]

**Supplemental Table 1.** Diagnosis codes used to identify patients with AUD-related admissions.

| **Description** | **ICD-10 Code** |
| --- | --- |
| Alcohol abuse | F10.1 |
| Alcohol abuse uncomplicated | F10.10 |
| Alcohol abuse with intoxication | F10.120-F10.129 |
| Alcohol abuse with withdrawal | F10.130-F10.139 |
| Alcohol-related disorder with alcohol-induced mood disorder | F10.14 |
| Alcohol abuse with alcohol induced psychotic disorder | F10.150-F10.159 |
| Alcohol abuse with other alcohol-induced disorder | F10.18 |
| Alcohol abuse with unspecified alcohol-induced disorder | F10.19 |
| Alcohol dependence | F10.2, F10.20, F10.220-F10.229 |
| Alcohol dependence with withdrawal | F10.230-F10.239 |
| Alcohol dependence with alcohol-induced psychotic disorder | F10.250-F10.259 |
| Alcohol dependence with other alcohol-induced disorders | F10.280-F10.282, F10.288 |
| Alcohol dependence with other alcohol-induced disorders with unspecified alcohol-induced disorders | F10.29 |
| Alcohol use, unspecified with intoxication | F10.920-F10.929 |
| Alcohol use, unspecified with withdrawal | F10.930-F10.939 |
| Alcohol use, unspecified with alcohol-induced psychotic disorder | F10.950-F10.959 |
| Alcohol use, with alcohol-induced persisting amnestic disorder | F10.96 |
| Alcohol use, with alcohol-induced persistent dementia | F10.97 |
| Alcohol use, unspecified with other alcohol-induced disorder | F10.98 |
| Alcohol use, with unspecified with alcohol-induced psychotic disorder, unspecified | F10.99 |
| Alcoholic polyneuropathy | G62.1 |
| Toxic effects of ethanol | T51.00-T51.9 |
| Alcohol-induced pseudo-Cushing Syndrome | E24.4 |
| Degeneration of nervous system due to alcohol | G31.2 |
| Alcoholic myopathy | G72.1 |
| Alcoholic cardiomyopathy | I42.6 |
| Alcoholic gastritis | K29.2 |
| Alcoholic liver disease | K70.0-K70.9 |
| Alcohol-induced acute pancreatitis | K85.2 |
| Alcohol-induced chronic pancreatitis | K86.0 |
| Maternal care for (suspected) damage to fetus from alcohol | O35.4 |
| Fetal alcohol syndrome (dysmorphic) | Q86.0 |
| Finding of alcohol in blood | R78.0 |
| Accidental poisoning by and exposure to alcohol | X45 |
| Intentional self-poisoning by and exposure to alcohol | X65 |
| Alcohol abuse counseling and surveillance | Z71.4 |
| Poisoning by and exposure to alcohol, undetermined intent | Y15 |
| Alcohol intoxication | Y91.0-Y91.3 |
| Alcohol involvement, not otherwise specified | Y91.9 |
| Problems related to lifestyle, alcohol use | Z72.1 |

**Supplemental Table 2.** Charlson and Elixhauser Comorbidity Index conditions present at first admission, *n* (%).

|  | **Rural Hospital Patients N=12,953 (%)** | **Urban Hospital Patients N=177,199 (%)** | **P-Value*** |
| --- | --- | --- | --- |
| Congestive Heart Failure | 336 (2.6) | 6765 (3.8) | <0.001 |
| Cardia arrhythmias | 610 (4.7) | 8402 (4.7) | 0.868 |
| Valvular disease | 90 (0.7) | 1478 (0.8) | 0.091 |
| Pulmonary circulation disorders | 64 (0.5) | 1300 (0.7) | 0.002 |
| Peripheral vascular disorders | 216 (1.7) | 3842 (2.2) | <0.001 |
| Hypertension, uncomplicated | 1493 (11.5) | 18992 (10.7) | 0.004 |
| Hypertension, complicated | 117 (0.9) | 1370 (0.8) | 0.105 |
| Paralysis | 14 (0.1) | 175 (0.1) | 0.745 |
| Other neurological disorders | 432 (3.3) | 5062 (1.9) | 0.002 |
| Chronic pulmonary disease | 837 (6.5) | 9694 (5.5) | <0.001 |
| Diabetes, uncomplicated | 383 (3.0) | 4918 (2.8) | 0.226 |
| Diabetes, complicated | 333 (2.6) | 4949 (2.8) | 0.138 |
| Hypothyroidism | 269 (2.1) | 2380 (1.3) | <0.001 |
| Renal failure | 203 (1.6) | 3941 (2.2) | <0.001 |
| Liver disease | 993 (7.7) | 14619 (8.3) | 0.020 |
| Peptic ulcer disease excluding bleeding | 19 (0.2) | 210 (0.1) | 0.372 |
| AIDS/HIV | 13 (0.1) | 461 (0.3) | <0.001 |
| Lymphoma | 23 (0.2) | 382 (0.2) | 0.365 |
| Metastatic cancer | 25 (0.2) | 1422 (0.8) | <0.001 |
| Solid tumor without metastasis | 284 (2.2) | 8091 (4.6) | <0.001 |
| Rheumatoid arthritis/collagen vascular disease | 30 (0.2) | 338 (0.2) | 0.307 |
| Coagulopathy | 150 (1.2) | 2103 (1.2) | 0.770 |
| Obesity | 330 (2.6) | 4522 (2.6) | 0.976 |
| Weight loss | 152 (1.2) | 2985 (1.7) | <0.001 |
| Fluid and electrolyte disorders | 388 (3.0) | 4742 (2.7) | 0.030 |
| Blood loss anemia | 36 (0.3) | 549 (0.3) | 0.527 |
| Deficiency anemia | 199 (1.5) | 3024 (1.7) | 0.147 |
| Alcohol abuse | 5539 (42.8) | 55445 (31.3) | <0.001 |
| Drug abuse | 1941 (15.0) | 26106 (14.7) | 0.434 |
| Psychoses | 410 (3.2) | 5257 (3.0) | 0.200 |
| Depression | 2740 (21.2) | 26970 (15.2) | <0.001 |
| None | 4317 (33.3) | 68313 (38.6) | <0.001 |

**Supplemental Table 3:** Outcomes and hospital characteristics of AUD-related admissions outcomes in Rural vs Urban VHA hospitals FY2016-FY2020 with observation admissions removed.

|  | Overall  (N=129) | Rural Hospital  (N=22) | Urban Hospital  (N=107) | P-Value* |
| --- | --- | --- | --- | --- |
| Total Alcohol related admissions (% of total admits) | 354769 (14.0) | 26422 (18.2) | 328347 (13.8) | <0.001 |
| Alcohol related admissions rate, Mean % (Std) | 15.5 (6.5) | 20.0 (9.8) | 14.5 (5.1) | <0.001 |
| 30-Day Mortality Rate, Mean % (Std) | 3.3 (1.5) | 2.3 (0.9) | 3.5 (1.5) | <0.001 |
| AverageYearly LOS in days, Median (IQR) | 5.8 (4.8-7.0) | 4.7 (3.8-5.7) | 6.1 (5.1-7.3) | <0.001 |
| 30-Day Readmission Rate, Mean %(Std) | 15.5 (4.1) | 15.4 (4.1) | 16.3 (4.3) | 0.041 |
| Treating specialty |  |  |  | <0.001 |
| Medicine | 202272 (57.0) | 18351 (69.5) | 183921 (56.0) |  |
| Psychiatry | 126553 (35.7) | 7832 (29.6) | 118721 (36.2) |  |
| Surgery | 25944 (7.3) | 239 (0.9) | 25705 (7.8) |  |
| Other | 0 (0.0) | 0 (0.0) | 0 (0.0) |  |
| Highest Level of Care |  |  |  | <0.001 |
| ICU | 25595 (7.2) | 1152 (4.4) | 24443 (7.4) |  |
| Inpatient | 329174 (92.8) | 25270 (95.6) | 303904 (92.6) |  |
| Observation | 0 (0.0) | 0 (0.0) | 0 (0.0) |  |
| Mortality rate for population with 1,3, and 5 years follow up |  |  |  |  |
| 1-year mortality | 20057/168579 (11.9) | 1146/11581 (9.9) | 18911/156998 (12.1) | <0.001 |
| 3-year mortality | 39588/168521 (23.5) | 2428/11578 (21.0) | 37160/156943 (23.7) | <0.001 |
| 5-year mortality | 38417/116139 (33.1) | 2572/8391 (30.7) | 35845/107748 (33.3) | <0.001 |
| * Categorical variables were assessed via Chi-square Test. Rates were assessed using the two-sample t-test, and medians were assessed via the Wilcoxon Rank Sum Test. All hypothesis tests were two-sided. | | | | |

**Supplemental Table 4:** Frailty Model (Cox proportional hazard model with additional covariates and clustering using a random intercepts for each hospital)

| **Variable** | **Hazard Ratio** | **95% CI** | **P-value** |
| --- | --- | --- | --- |
| First admission age | 1.051 | 1.050, 1.052 | <0.001 |
| Hospital Rurality (ref=urban) | 0.995 | 0.932, 1.061 | 0.871 |
| Sex (ref=male) | 0.705 | 0.672, 0.740 | <0.001 |
| Rural Patient (ref=urban) | 0.967 | 0.949, 0.985 | <0.001 |
| Missing rurality patient (ref=urban) | 0.713 | 0.525, 0.969 | 0.031 |
| American Indican or Alaskan Native Race (ref=White) | 1.113 | 1.037, 1.194 | <0.001 |
| Asian or Pacific Islander Race (ref=White) | 0.915 | 0.844, 0.993 | 0.033 |
| Black Race (ref=White) | 0.814 | 0.798, 0.830 | <0.001 |
| Unknown Race (ref=White) | 0.954 | 0.923, 0.986 | 0.005 |

**Supplemental Table 5:** Mortality of patients with first admission during COVID-19 pandemic.

| Mortality among those with first admission between 3/1/20-9/30/20 | | | | |
| --- | --- | --- | --- | --- |
|  | Overall | Rural | Urban | p-value |
| Year 1 Mortality | 1829/14229 (12.9%) | 93/869 (10.7%) | 1736/13360 (13.0%) | 0.050 |
| Year 3 Mortality | 3436/14159 (24.3%) | 211/865 (24.4) | 3225/13294 (24.3%) | 0.929 |
